# Rapid Symptom Resolution After Adjunctive Oral Colloidal Silicon Dioxide in a Non-anaphylactic Hazelnut Allergy Reaction: A Case Report

**DOI:** 10.7759/cureus.117900

**Published:** 2026-10-14

**Authors:** Tahir Sahin, Muhammed B Sarihan

**Affiliations:** 1 Emergency Department, Ankara Etlik City Hospital, Ankara, TUR; 2 Department of Emergency Medicine, Sincan Training and Research Hospital, University of Health Sciences, Ankara, TUR

**Keywords:** colloidal silicon dioxide, emergency department, enterosorbent, food allergy, hazelnut, urticaria

## Abstract

Acute food allergy reactions that do not fulfil the criteria for anaphylaxis are common in the emergency department and are managed symptomatically, principally with H1 antihistamines. No current recommendation addresses the allergen that remains unabsorbed in the gastrointestinal lumen at the time of presentation.

We report a case of a 44-year-old man with childhood-onset hazelnut allergy who presented 30 minutes after inadvertently eating a packaged dessert containing hazelnut, with generalised urticaria, intense pruritus, nausea, vomiting and crampy abdominal pain. He was haemodynamically stable with no respiratory or laryngeal involvement and did not meet diagnostic criteria for anaphylaxis, so adrenaline was not given. Alongside standard care with an intravenous H1 antihistamine and intravenous fluids, a single 3 g oral dose of colloidal silicon dioxide (SiO_2_), a non-absorbed silica-based enterosorbent, was administered. Fifty minutes later, his gastrointestinal symptoms had resolved and the urticaria was found fading. He was observed for eight hours, developed no biphasic reaction, and was discharged uneventfully.

Adjunctive enterosorbent therapy was well tolerated and was followed by rapid improvement. Because an intravenous antihistamine was given concurrently and spontaneous resolution cannot be excluded, no causal role can be attributed to the enterosorbent. The case is reported to draw attention to a mechanistically plausible but untested adjunct, not to advocate its use.

## Introduction

Tree nut allergy is among the most common causes of IgE-mediated food reactions in adults, and hazelnut is a frequent trigger in European populations. Most emergency presentations are not anaphylaxis but acute multi-system reactions confined to the skin and gastrointestinal tract. For these, the 2025 European Academy of Allergy and Clinical Immunology (EAACI) guideline on the management of IgE-mediated food allergy recommends symptomatic treatment with H1 antihistamines, reserving adrenaline for reactions meeting anaphylaxis criteria [1].

Antihistamines act on histamine already released at the receptor. They do not affect allergens that are still in the gut and still being absorbed. Because gastric emptying of solid food takes 2-4 hours in adults [2], a patient presenting within the first hour of ingestion may still harbour unabsorbed allergen, and hazelnut storage protein allergens such as Cor a 9 and Cor a 14 are notably resistant to heat and peptic digestion [3]. Reducing this intraluminal load is therefore a theoretically attractive but unaddressed target.

Colloidal silicon dioxide (SiO_2_) is a highly dispersed inorganic enterosorbent that is neither absorbed nor metabolised and binds antigens, bacterial toxins, and low-molecular-weight compounds within the gut lumen [4]. Its strongest evidence is in acute diarrhoea, where a randomised, double-blind, placebo-controlled trial in 144 patients showed a significant reduction in symptom duration [5]. In food allergy, an open randomised study in children reported faster symptom resolution when the agent was added to baseline therapy for 10-14 days [6]. We are not aware of a published report of single-dose use in an adult presenting acutely.

This case report was prepared and reported in accordance with the CAse REport (CARE) guidelines [7].

## Case presentation

A 44-year-old man presented to the emergency department with a generalised itchy rash, nausea, vomiting and abdominal pain. He had a hazelnut allergy diagnosed in childhood and had had two previous accidental exposures, both causing urticaria and lip tingling that settled with oral antihistamines; neither had required adrenaline or admission. His past medical and surgical history was otherwise unremarkable; he had no chronic systemic, cardiovascular, or gastrointestinal diseases, no asthma, no atopic dermatitis, and no other food or drug allergy, and he took no regular medication. An adrenaline auto-injector had been prescribed years earlier but was not being carried.

Thirty minutes before arrival, he had eaten two pieces of a commercially packaged dessert at a family gathering. Palmar and scalp pruritus began about 15 minutes after ingestion and progressed over the next 10 minutes to a generalised rash with nausea. A relative examined the packaging during the visit and confirmed hazelnut among the declared ingredients. He had no known allergy to any other food, and the clinical features closely resembled his two previous reactions to hazelnut. The other foods served at the gathering were not documented, so a contribution from another allergen cannot be entirely excluded. He had taken no medication before arrival.

On arrival, he was alert, oriented, anxious, and visibly uncomfortable but speaking in full sentences. Blood pressure was 128/78 mmHg, heart rate 96 beats/min, respiratory rate 18/min, oxygen saturation 98% on room air, and temperature 36.7°C. There were widespread, partly confluent urticarial wheals over the trunk, both upper limbs, and anterior thighs, covering an estimated 40% of the body surface, with marked dermographism. The abdomen was soft with diffuse mild periumbilical tenderness and increased bowel sounds, without guarding or rebound.

Respiratory and cardiovascular involvement was absent throughout. There was no dyspnoea, stridor, hoarseness, or throat tightness; the chest was clear with no wheeze; there was no lip, tongue, or uvular angioedema; and there was no hypotension, syncope, or altered consciousness. Gastrointestinal involvement consisted of nausea, vomiting that did not recur beyond 10 minutes after arrival, and crampy abdominal pain with only mild periumbilical tenderness and no guarding; all gastrointestinal symptoms had resolved by 70 minutes. The World Allergy Organization (WAO) 2020 criteria include severe gastrointestinal symptoms alongside skin involvement, but this criterion refers especially to non-food allergens [8], and the gastrointestinal features in this case were neither severe nor sustained. He therefore did not fulfil the WAO or EAACI criteria for anaphylaxis [1,8], and intramuscular adrenaline was not indicated. This assessment was documented, and he was placed on continuous monitoring so that it could be revisited if his condition changed.

Blood tests showed a white cell count of 9.4 × 10^9^/L (reference range: 4.5-10 × 10^9^/L), eosinophils 0.28 × 10^9^/L (reference range: 0-0.5 × 10^9^/L), C-reactive protein 3.1 mg/L (reference range: 0-5 mg/L), and normal renal function and electrolytes. Serum tryptase obtained 60 minutes after symptom onset was 5.8 µg/L (reference range: < 11.4 µg/L). As tryptase remains within the normal range in a substantial proportion of anaphylaxis episodes, including food-induced reactions [8], this result was considered non-contributory, and the classification rested on clinical criteria alone. Acute gastroenteritis was considered unlikely in an afebrile patient with normal inflammatory markers and prominent urticaria; scombroid poisoning was excluded because the food was a packaged dessert and no other diner became unwell; and a drug reaction was excluded as no medication had been taken.

Standard treatment was started 10 minutes after arrival with intravenous pheniramine maleate 45.5 mg given slowly, together with 500 mL of 0.9% sodium chloride. No corticosteroid was given. Since ingestion had occurred only 30 minutes earlier and gastrointestinal symptoms were prominent, a substantial residual allergen load was thought likely to remain in the stomach and proximal duodenum. After the rationale, the off-label nature of the intervention and the alternatives were explained and verbal consent was obtained; a single 3 g dose of colloidal SiO_2_ powder (Polisorb MP; JSC Polisorb, Kopeysk, Russia) suspended in approximately 100 mL of water was given orally 20 minutes after arrival. According to the manufacturer, the product consists of highly dispersed silica with a particle size of up to 0.09 mm and a specific surface area of approximately 300 m^2^/g. The 3 g dose corresponds to a single standard adult dose (approximately one heaped tablespoon of powder), in line with the manufacturer's recommended average adult daily dose of 6-12 g given in three to four divided doses. Vomiting had ceased, and the airway was unequivocally secure before administration. The complete clinical timeline is summarised in Table 1.

**Table 1:** Clinical timeline BSA: Body surface area; IV: Intravenous; NaCl: Sodium chloride; VAS: Visual analogue scale

Time	Event	Clinical status
-30 min	Ingestion of packaged dessert containing hazelnut	Asymptomatic
0	Emergency department arrival	Urticaria ~40% BSA, pruritus VAS 8/10, nausea, vomiting, abdominal pain
+10 min	IV pheniramine maleate 45.5 mg; 500 mL 0.9% NaCl	One further episode of vomiting; airway clear
+20 min	Oral colloidal silicon dioxide 3 g in 100 mL water	Suspension tolerated; no further vomiting
+70 min	Reassessment, 50 min after enterosorbent	Nausea and abdominal pain resolved; wheals fading; pruritus VAS 3/10
+4 h	Interim reassessment	Faint macular erythema only; pruritus VAS 1/10
+8 h	Observation completed, discharged	Asymptomatic; no biphasic reaction

Fifty minutes after the enterosorbent - 70 minutes after arrival - the nausea and abdominal pain had resolved; there was no further vomiting; the wheals were fading into faint macular erythema; and pruritus had fallen from 8/10 to 3/10 on the visual analogue scale (VAS). Vital signs remained stable throughout.

He was observed for eight hours. This was longer than strictly necessary for a reaction of this severity and reflected the prominent gastrointestinal involvement, the fact that he was not carrying an adrenaline auto-injector, and the use of an off-label adjunct. No biphasic reaction occurred and no adverse effect attributable to the enterosorbent - constipation, distension, or vomiting - was seen. He was discharged on cetirizine 10 mg daily for five days with written hazelnut and tree nut avoidance advice, including guidance on precautionary allergen labelling, a new adrenaline auto-injector prescription with trainer-device instruction, a written emergency action plan, and referral to the allergy clinic for component-resolved diagnostics and baseline tryptase. He was asymptomatic at telephone follow-up on day 7 and attended his clinic appointment.

## Discussion

This patient improved rapidly after a single dose of an oral enterosorbent given alongside standard treatment for a non-anaphylactic hazelnut reaction, and the intervention was well tolerated. The interest of the case lies in the timing: he presented 30 minutes after eating a solid food, and we hypothesised that much of the ingested allergen was still in the stomach and proximal small bowel at that point [2]. This remains an assumption, as no luminal or circulating allergen was measured. A non-selective sorbent with a large adsorptive surface would, in principle, be able to bind such protein intraluminally, acting at a different point in the pathway from an antihistamine, which antagonises mediators that have already been released. Increased intestinal permeability is recognised to facilitate allergen access to the submucosa and to drive the T helper type 2 (Th2) response underlying food allergy [9], and reducing the luminal antigen available for absorption is a plausible way to attenuate that flux.

Plausibility, however, is not evidence, and the limitations of this observation are decisive. The patient received intravenous pheniramine and intravenous fluid 10 minutes before the enterosorbent. Intravenous H1 antihistamines act within minutes, and improvement at 70 minutes after arrival is fully explained by that treatment alone; this remains the most parsimonious interpretation. Moreover, spontaneous resolution of the reaction cannot be excluded, so the observed trajectory is also compatible with natural history. In this case, no measurement of luminal or circulating allergen was made, so the proposed mechanism was not tested, and pruritus was assessed by a subjective VAS rather than a standardised scoring system such as the urticaria activity score (UAS) [10]. Allergen attribution rests on history and label review rather than oral challenge.

The published evidence for colloidal SiO_2_ does not close this gap. A randomised, double-blind trial in acute diarrhoea establishes that the compound has genuine intraluminal activity under blinded conditions [5], but it is a different indication. The paediatric food allergy data are open-label and unblinded with subjective endpoints and describe a 10-14 day maintenance course rather than a single acute dose [6]; a recent review of enterosorbents in paediatric food allergy sets out the rationale while acknowledging that most supporting studies are of limited methodological quality [4]. Whether an agent that may reduce cumulative antigen exposure over days behaves the same way as a single dose given acutely is an open question that this case cannot answer.

Several practical points deserve emphasis. Enterosorbents are non-selective and may reduce absorption of concurrently given oral drugs, with an interval of at least one hour advised; where an oral antihistamine is the mainstay of treatment, this is a substantive argument against concurrent use, and in our patient, the antihistamine was given intravenously. An oral suspension must never be given to a patient with active vomiting, impaired consciousness, progressive oropharyngeal oedema, or any doubt about airway protection. Suspected gastrointestinal obstruction, bleeding, or ulceration are contraindications. Because the use is off-label, the rationale and the uncertainty of benefit should be explained and consent documented.

Most importantly, nothing here modifies established practice. The EAACI guideline makes no provision for enterosorbents in acute reaction management [1]. In this case, the patient was formally assessed against anaphylaxis criteria before any adjunct was considered; the patient would have received intramuscular adrenaline immediately had those criteria been met at any point. He received the enterosorbent after, never instead of, the standard therapy and was discharged with every guideline-mandated component of care. Enterosorbents have no role in anaphylaxis, where delay to adrenaline is associated with worse outcomes.

The question raised by this case is answerable. A double-blind, placebo-controlled trial in adults presenting within two hours of ingestion with a non-anaphylactic food allergy reaction, comparing an enterosorbent with placebo added to standard antihistamine therapy, with time to symptom resolution as the primary endpoint, would settle it. Until such data exist, the observation reported here should be regarded as hypothesis-generating only.

## Conclusions

In this adult with an acute, non-anaphylactic hazelnut allergy reaction confined to the skin and gastrointestinal tract, a single dose of oral colloidal SiO_2_ added to standard intravenous antihistamine therapy was well tolerated and was followed by marked improvement within 50 minutes and uneventful discharge after eight hours. No causal contribution can be attributed to the enterosorbent, and its use should not be adopted on the basis of this report. Reducing the intraluminal allergen load in patients who present soon after ingestion is a plausible adjunctive strategy that warrants formal evaluation.
